# Supplementary material for: Development of a Versatile Lipid Core for Nanostructured Lipid Carriers (NLCs) Using Design of Experiments (DoE) and Raman Mapping
Source: Pharmaceutics. 2024 Feb 8;16(2):250. doi: 10.3390/pharmaceutics16020250 (PMC10893334; doi:10.3390/pharmaceutics16020250)
Supplement: Supplementary file 1 [file pharmaceutics-16-00250-s001.zip › pharmaceutics-2822877-supplementary.pdf]

pharmaceutics

MDPI

Article

Supplementary Materials: Development of a Versatile Lipid Core for Nanostructured Lipid Carriers (NLCs) Using Design of Experiments (DoE) and Raman Mapping

Carlos Alberto Rios, Roberta Ondei and Márcia Cristina Breitkreitz

Table S1. Drugs and (% w/w) used in the preparation of tablets

Drug	Mixture (% w/w)	Mass of drugs (mg)	Mass tablet (g)	LogP
Caffeine	10.0	300.0	3.0	-0.070
Acetaminophen	10.0	300.0		0.910
Butamben	5.0	150.0		2.870
Resveratrol	3.0	90.0		3.100
Tacrolimus	1.0	30.0		3.300
Atorvastatin calcium	10.0	300.0		6.360
Coenzyme Q10	3.0	90.0		10.000

Table S2. Classification of excipients according to behavior in water

	Excipients analyzed		
	**Hydrophobic**	**Medium Polarity**	**Hydrophilic**
**Liquid**	(1) SR™ Sesame Oil<br>(2) SR™ Soybean Oil<br>(3) SR™ Oleic Acid<br>(4) SR™ GTCC	(6) SR™ Lauryl Lactate<br>(7) SR™ PGMC	(9) SR™ DMI<br>(10) SR™ PEG 400<br>(11)SR™ Propylene Glycol
**Solid**	(5) Crodamol™ CP pharma	(8) SR™ GMCC **(S)**	(12) Croduret™ 40 **(S)**<br>(13) Crodasol™ HS HP **(S)**

SR™ = Super Refined™ , S= Surfactant

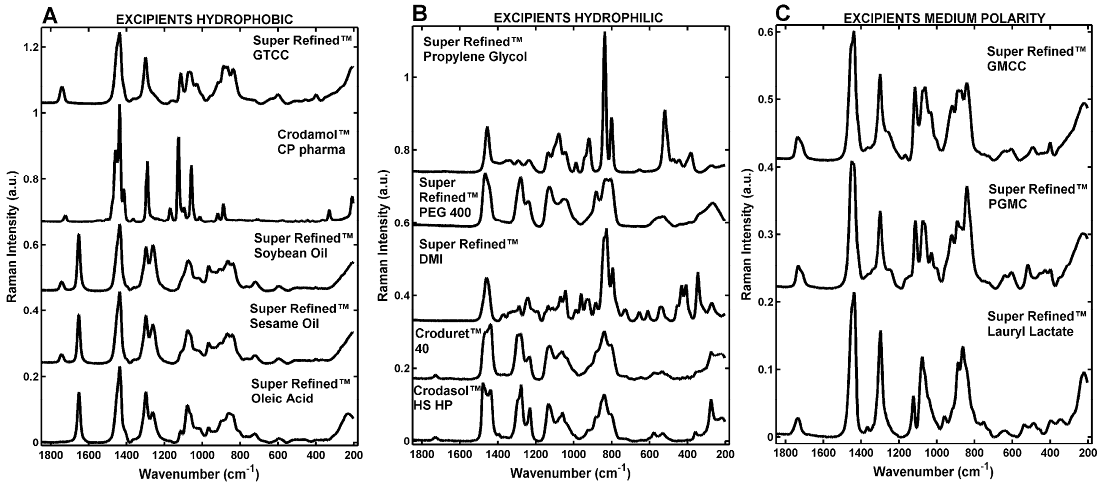

Figure S1. Raman spectrum of pure excipients that were classified as (A) hydrophobic, (B) hydrophilic and (C) medium polarity

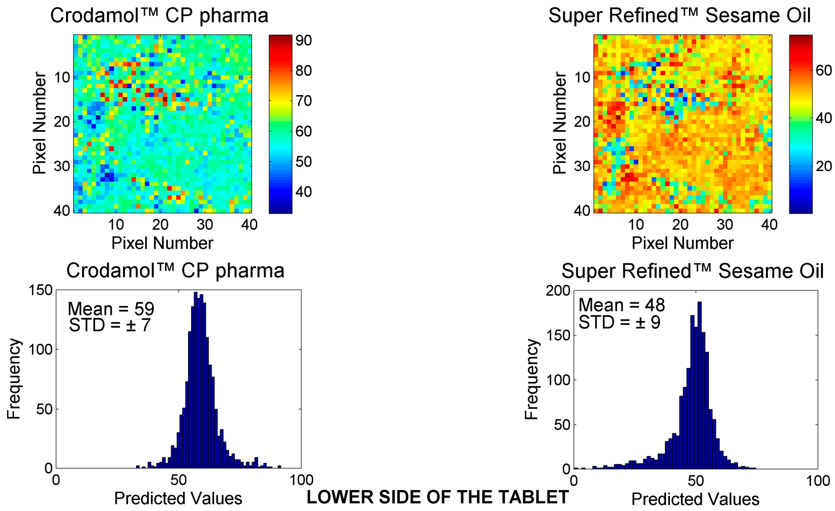

Figure S2. Chemical maps (replicate 1) obtained from the mixture of Crodamol™ CP pharma with Super Refined™ Sesame Oil in the 1:1 w/w ratio

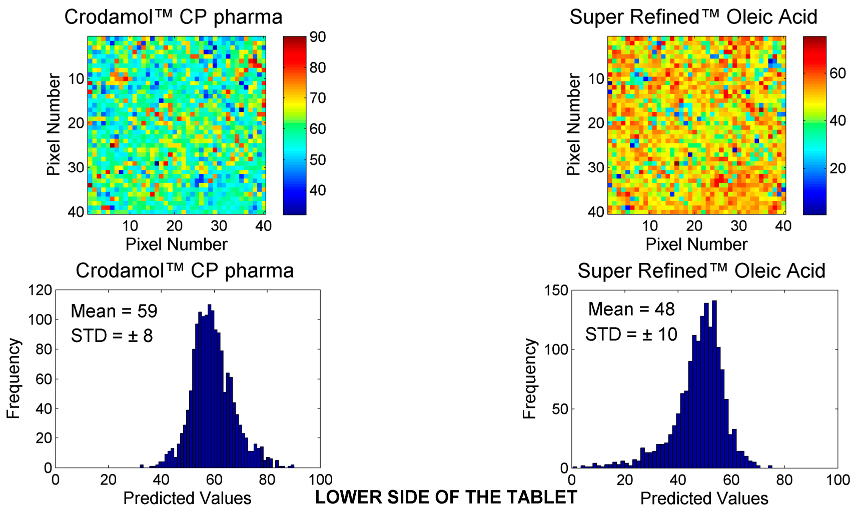

Figure S3. Chemical maps (replicate 1) obtained from the mixture of Crodamol™ CP pharma with Super Refined™ Oleic Acid in the 1:1 w/w ratio

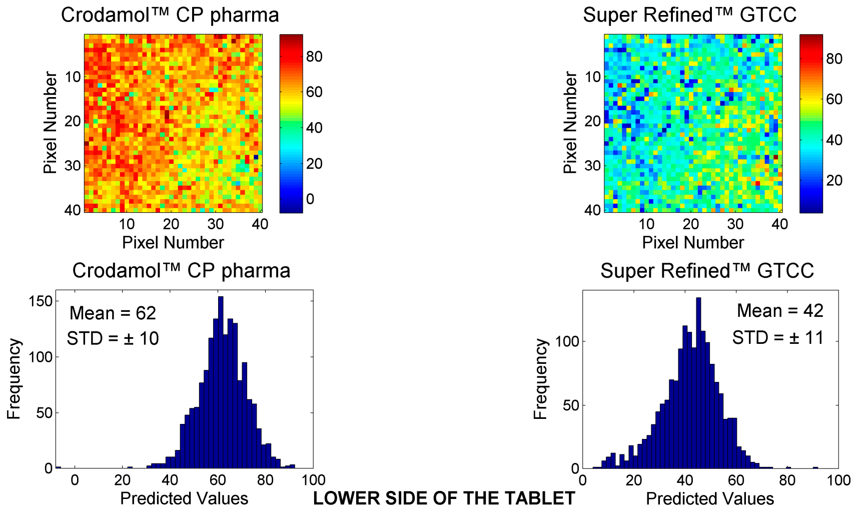

Figure S4. Chemical maps (replicate 1) obtained from the mixture of Crodamol™ CP pharma with Super Refined™ GTCC in the 1:1 w/w ratio

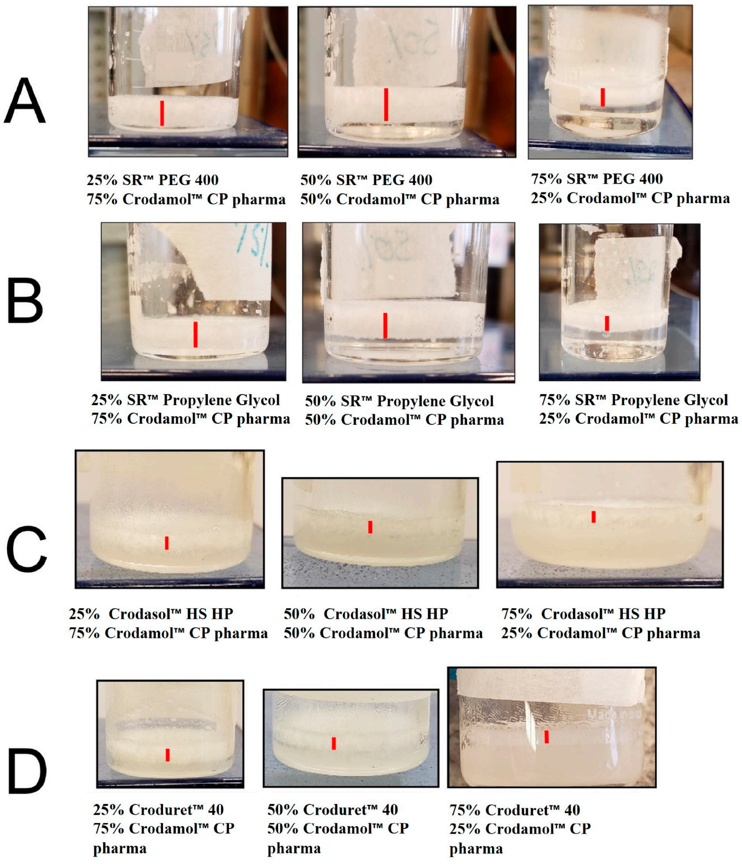

Figure S5. Preparation of tablets for excipients (A) Super Refined™ PEG 400, (B) Super Refined™ Propylene Glycol, (C) Crodasol™ HS HP, (D) Croduret™ 40 in proportions 1:1, 1:3 and 3:1 w/w

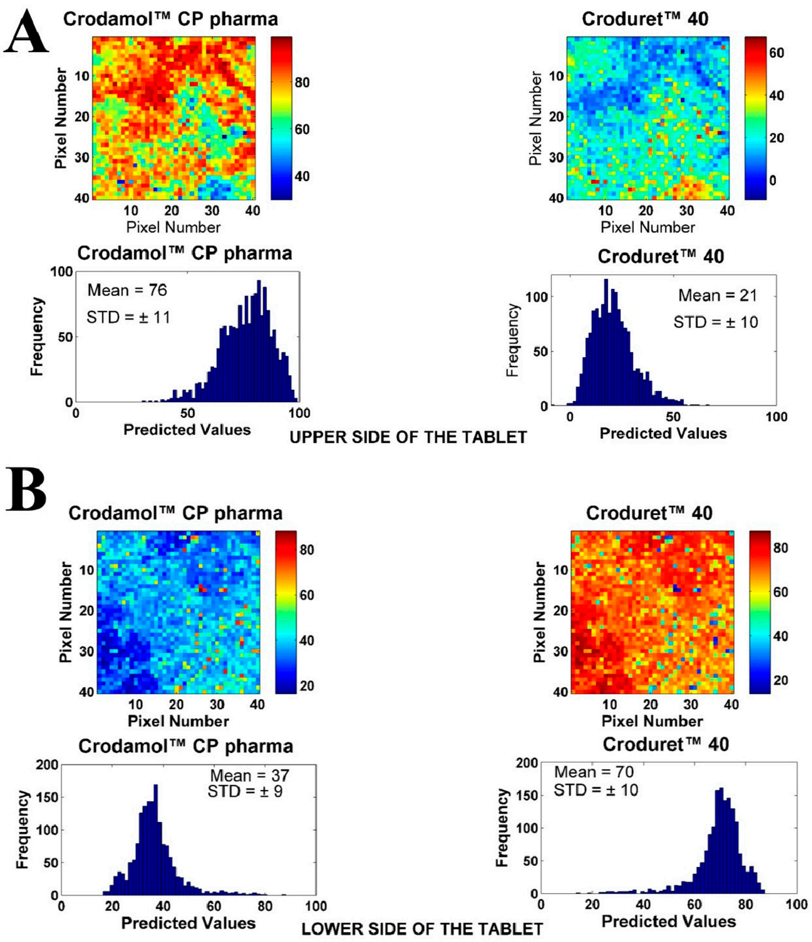

Figure S6. Chemical maps (replicate 1) obtained from (A) upper and (B) lower side of mixture between Crodamol™ CP pharma with Croduret™ 40 (in the 1:1 w/w ratio)

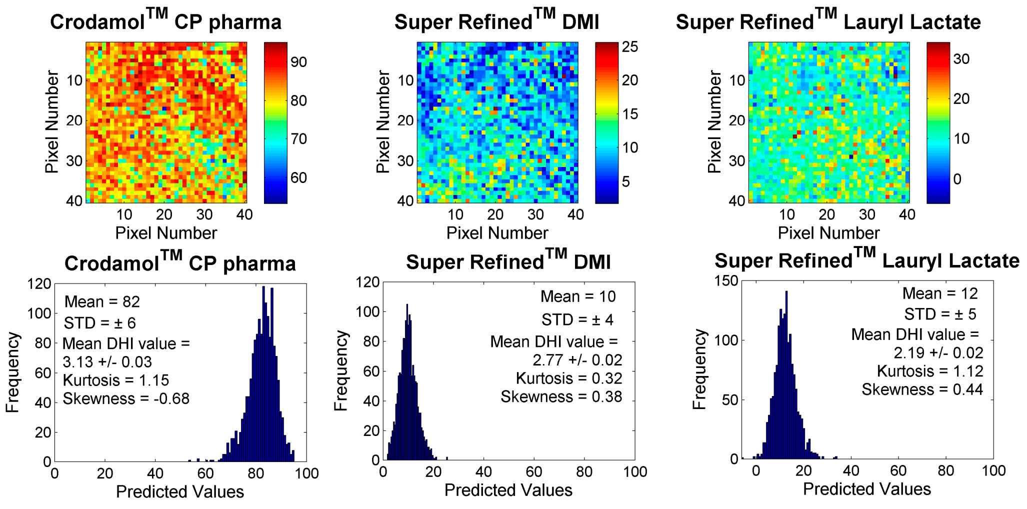

Figure S7. Chemical maps obtained from mixture design, Crodamol™ CP pharma (70% w/w), Super Refined™ DMI (10% w/w) and Super Refined™ Lauryl Lactate (10% w/w), replicate 1, point A

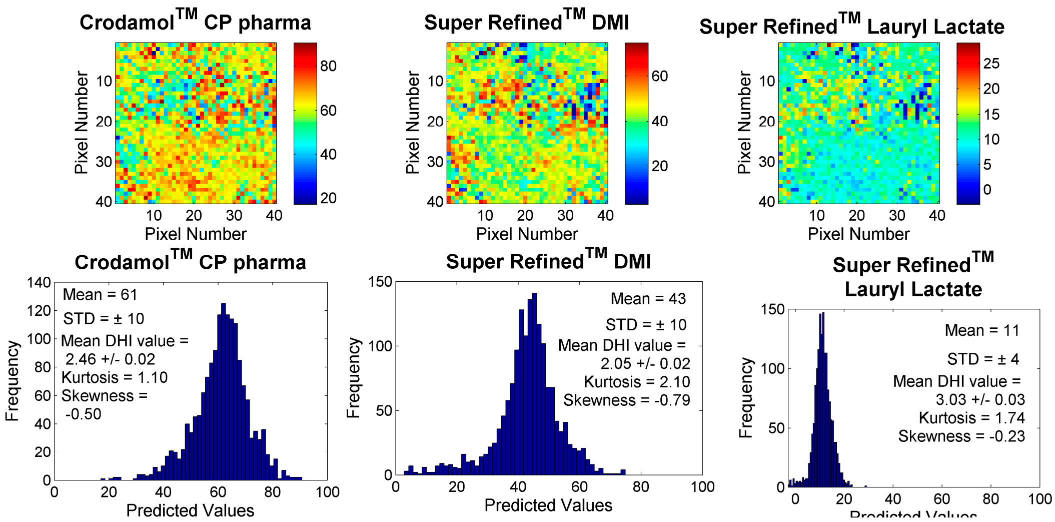

Figure S8. Chemical maps obtained from mixture design, Crodamol™ CP pharma (40% w/w), Super Refined™ DMI (40% w/w) and Super Refined™ Lauryl Lactate (10% w/w), replicate 1, point B

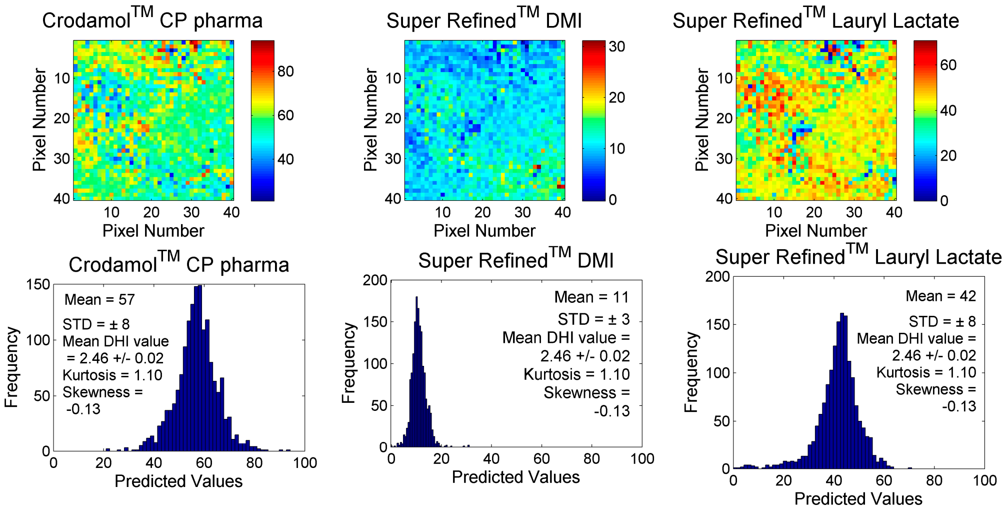

Figure S9. Chemical maps obtained from mixture design, Crodamol™ CP pharma (40% w/w), Super Refined™ DMI (10% w/w) and Super Refined™ Lauryl Lactate (40% w/w), replicate 1, point C

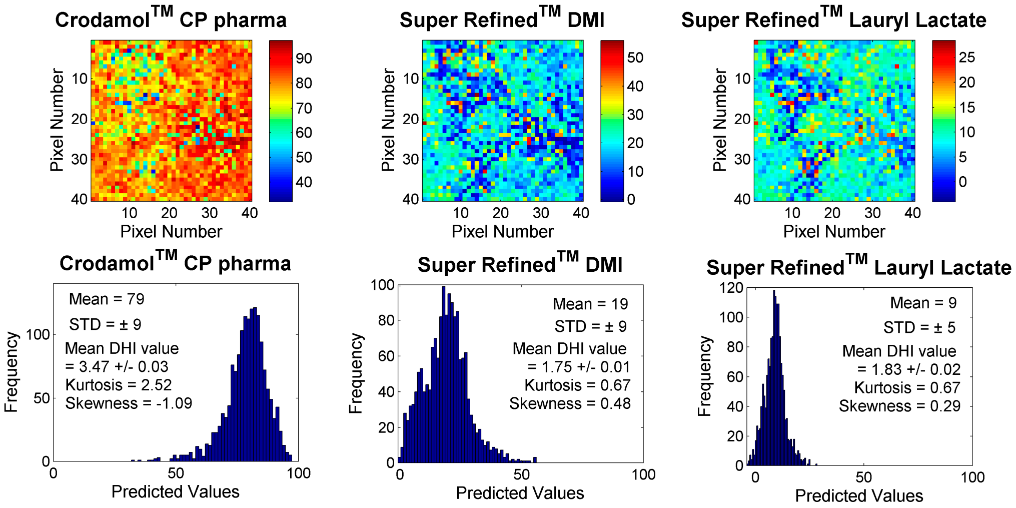

Figure S10. Chemical maps obtained from mixture design, Crodamol™ CP pharma (55% w/w), Super Refined™ DMI (25% w/w) and Super Refined™ Lauryl Lactate (10% w/w), point D

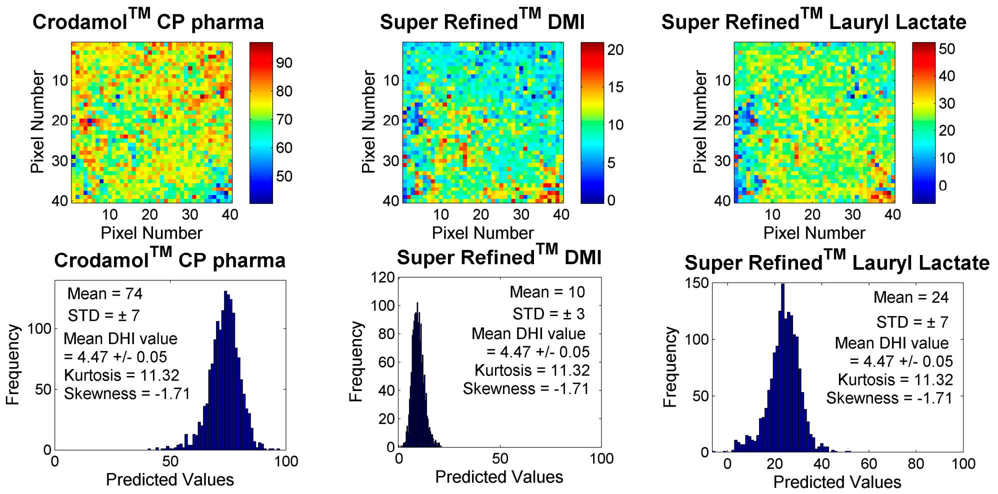

Figure S11. Chemical maps obtained from mixture design, Crodamol™ CP pharma (55% w/w), Super Refined™ DMI (10% w/w) and Super Refined™ Lauryl Lactate (25% w/w), point E

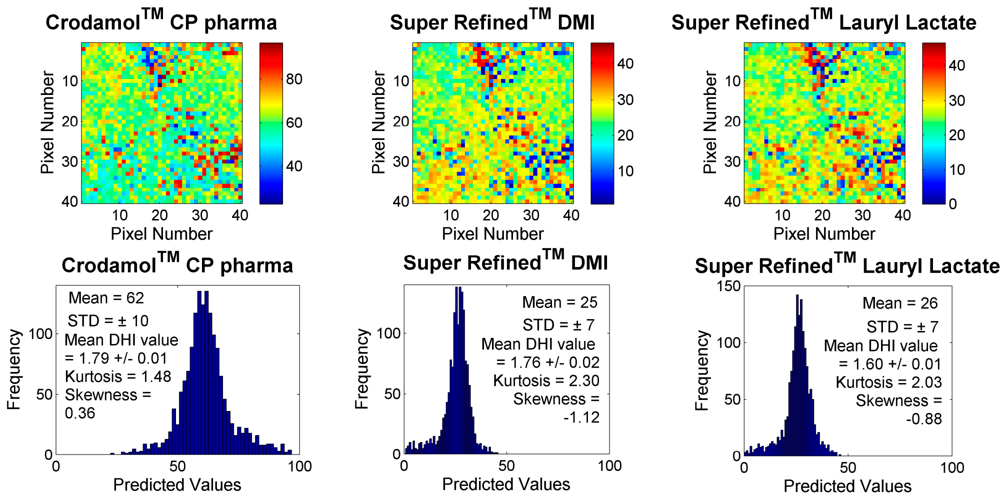

Figure S12. Chemical maps obtained from mixture design, Crodamol™ CP pharma (40% w/w), Super Refined™ DMI (25% w/w) and Super Refined™ Lauryl Lactate (25% w/w), point F

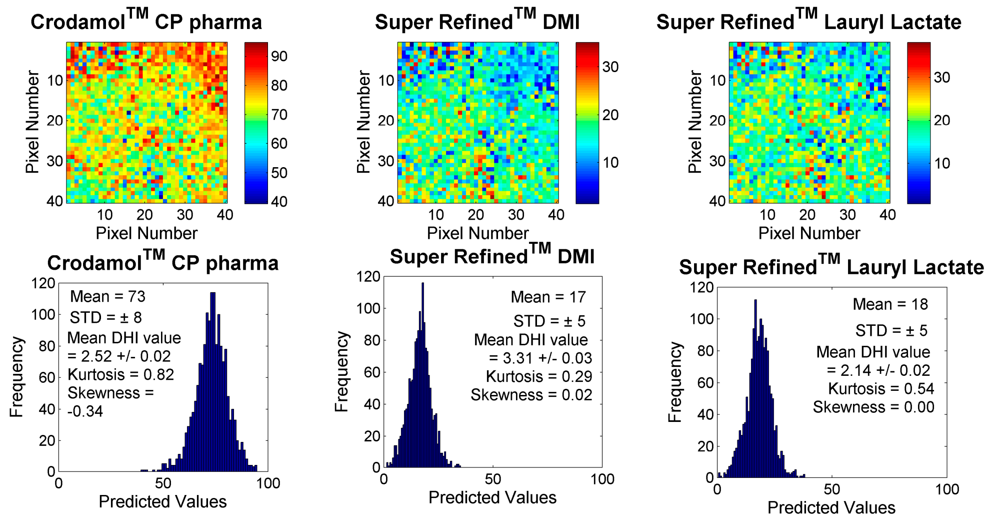

Figure S13. Chemical maps obtained from mixture design, Crodamol™ CP pharma (60% w/w), Super Refined™ DMI (15% w/w) and Super Refined™ Lauryl Lactate (15% w/w), point G

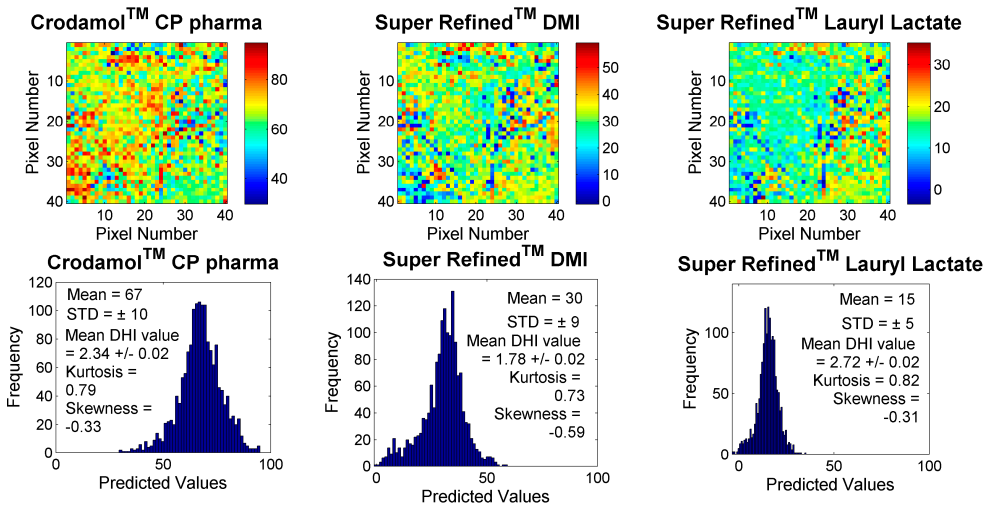

Figure S14. Chemical maps obtained from mixture design, Crodamol™ CP pharma (45% w/w), Super Refined™ DMI (30% w/w) and Super Refined™ Lauryl Lactate (15% w/w), point H

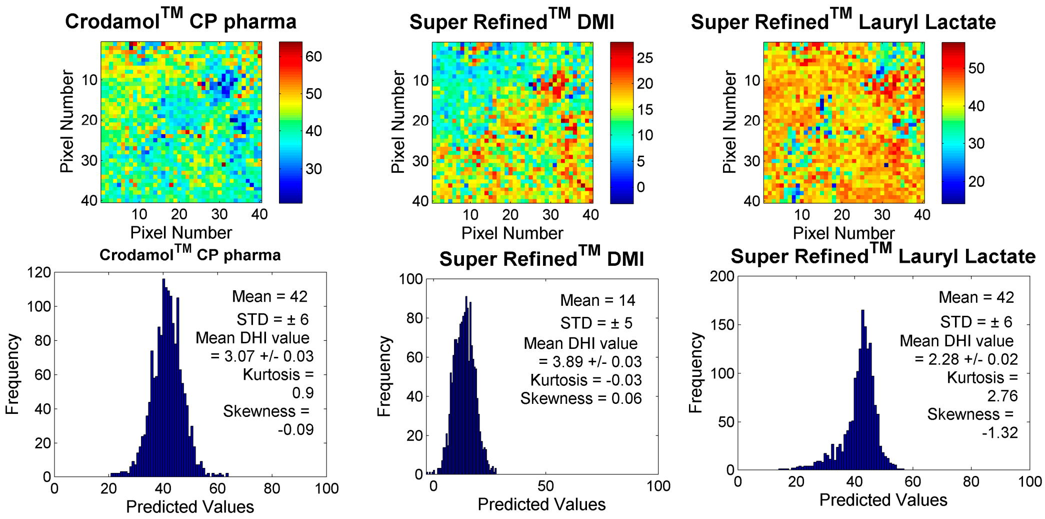

Figure S15. Chemical maps obtained from mixture design, Crodamol™ CP pharma (45% w/w), Super Refined™ DMI (15% w/w) and Super Refined™ Lauryl Lactate (30% w/w), point I

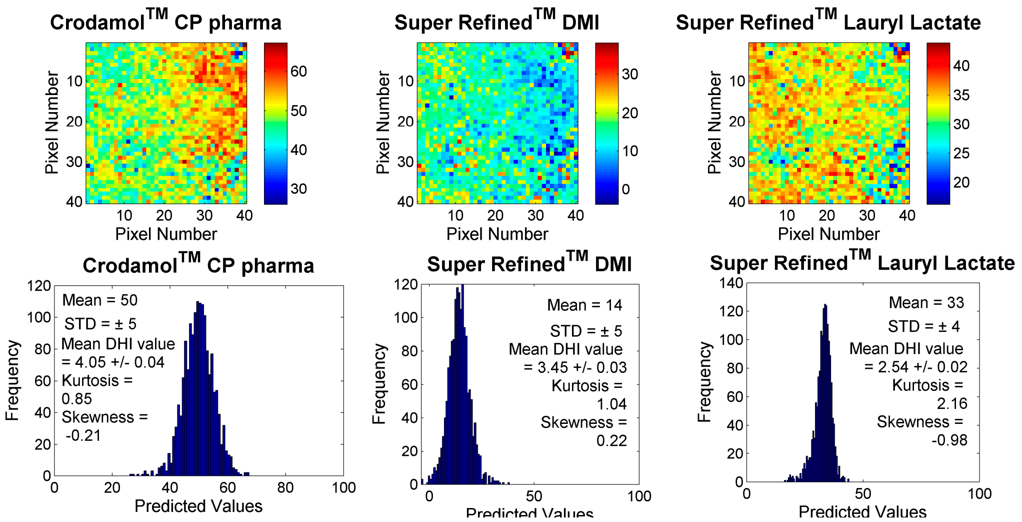

Figure S16. Chemical maps obtained from mixture design, Crodamol™ CP pharma (50% w/w), Super Refined™ DMI (20% w/w) and Super Refined™ Lauryl Lactate (20% w/w), replicate 1, point J

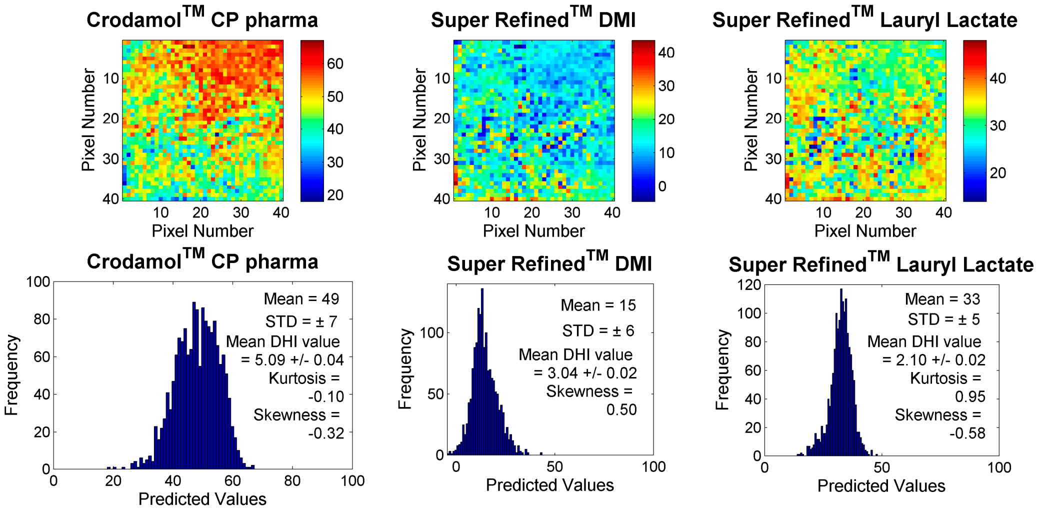

Figure S17. Chemical maps obtained from mixture design, Crodamol™ CP pharma (50% w/w), Super Refined™ DMI (20% w/w) and Super Refined™ Lauryl Lactate (20% w/w), replicate 2, point J

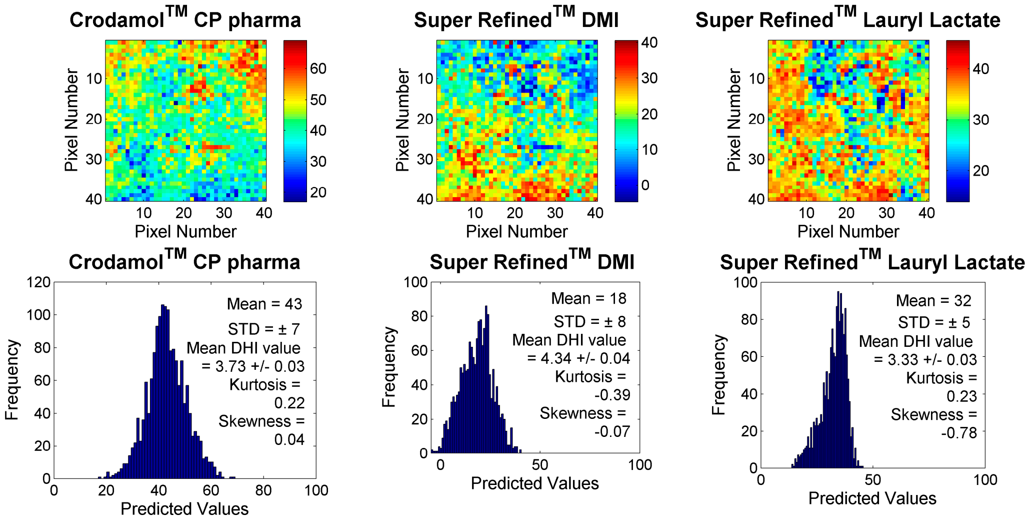

Figure S18. Chemical maps obtained from mixture design, Crodamol™ CP pharma (50% w/w), Super Refined™ DMI (20% w/w) and Super Refined™ Lauryl Lactate (20% w/w), replicate 3, point J

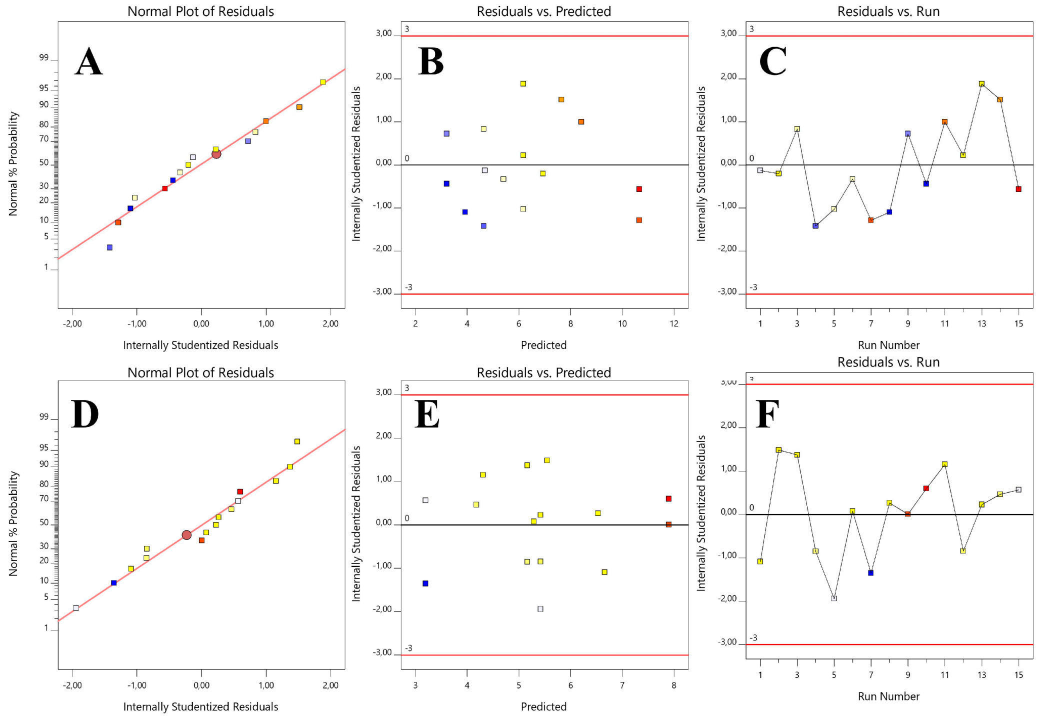

Figure S19. (A,D) Normal plot, (B,E) residuals vs. predicted and (C,F) residuals vs. run graph for responses Y_2 (A, B and C) and Y_3 (D, E and F)

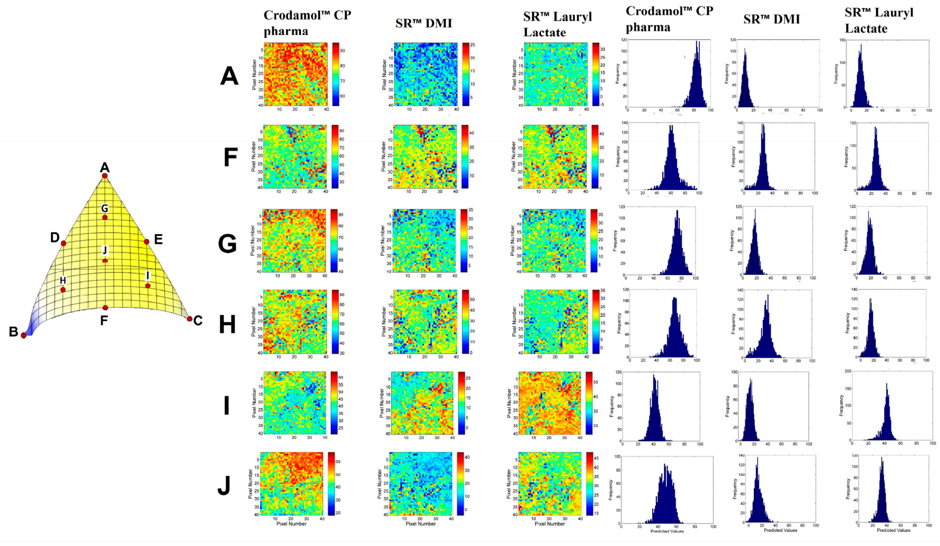

Figure S20. Chemical maps and histograms for the mixture design points A, F, G, H, I and J

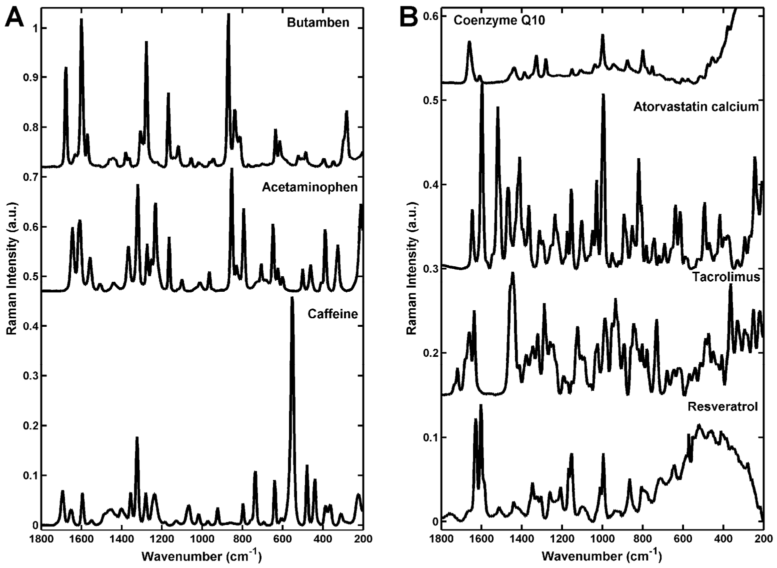

Figure S21. Raman spectra of the drugs (A) butamben, caffeine, acetaminophen and (B) coenzyme Q10, atorvastatin calcium, tacrolimus and resveratrol used in the study

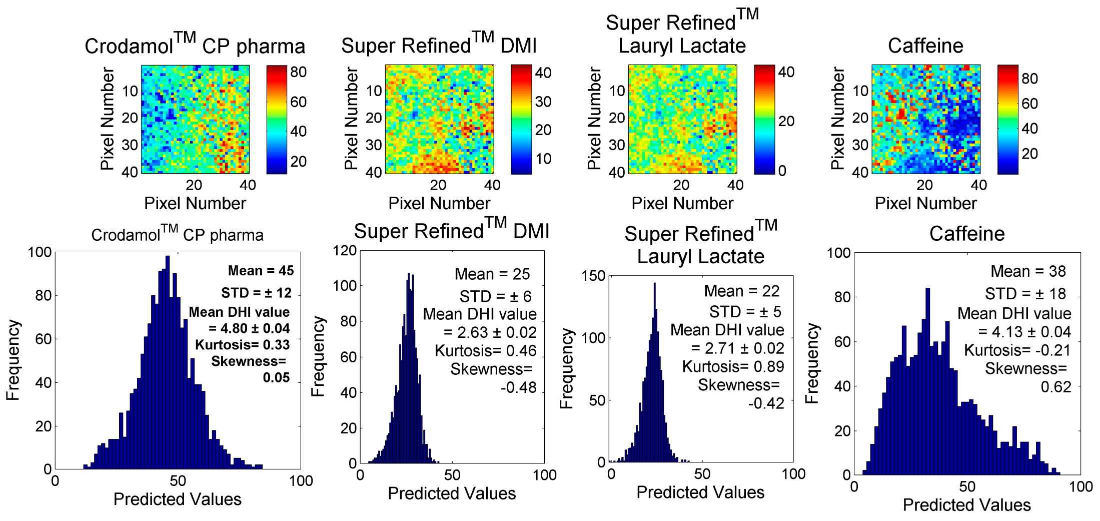

Figure S22. Chemical maps of the mixtures with the excipients Crodamol™ CP pharma (40 (% w/w)), SR™ DMI (25 (% w/w)) and SR™ Lauryl Lactate (25 (% w/w)) with the drug Caffeine, replicate 1

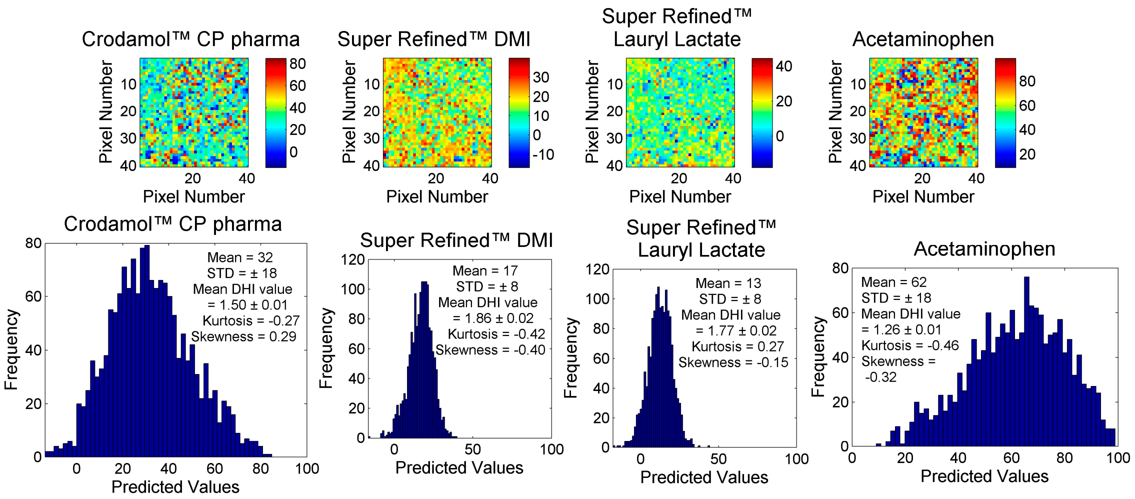

Figure S23. Chemical maps of the mixtures with the excipients Crodamol™ CP pharma (40 (% w/w)), SR™ DMI (25 (% w/w)) and SR™ Lauryl Lactate (25 (% w/w)) with the drug Acetaminophen, replicate 1

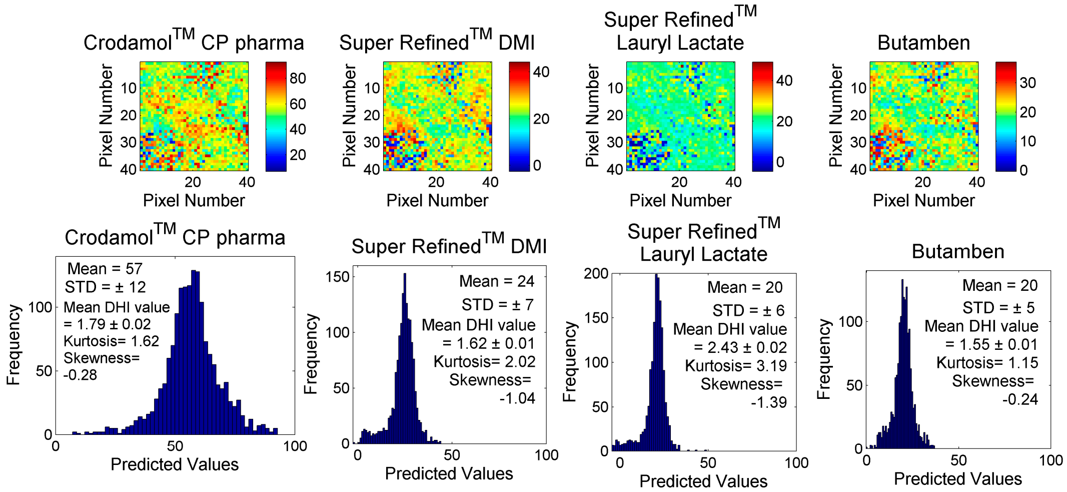

Figure S24. Chemical maps of the mixtures with the excipients Crodamol™ CP pharma (40 (% w/w)), SR™ DMI (25 (% w/w)) and SR™ Lauryl Lactate (25 (% w/w)) with the drug Butamben, replicate 1

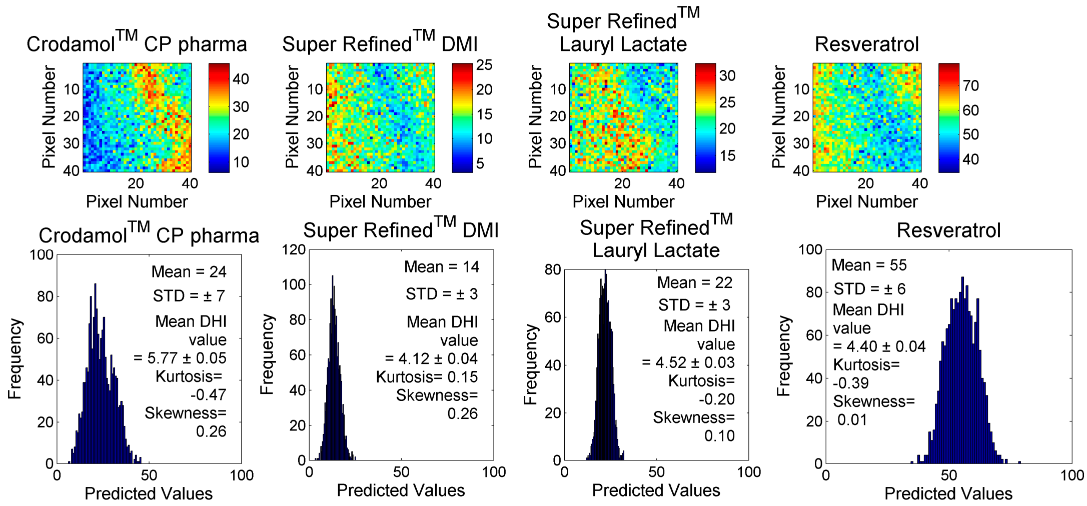

Figure S25. Chemical maps of the mixtures with the excipients Crodamol™ CP pharma (40 (% w/w)), SR™ DMI (25 (% w/w)) and SR™ Lauryl Lactate (25 (% w/w)) with the drug Resveratrol, replicate 1

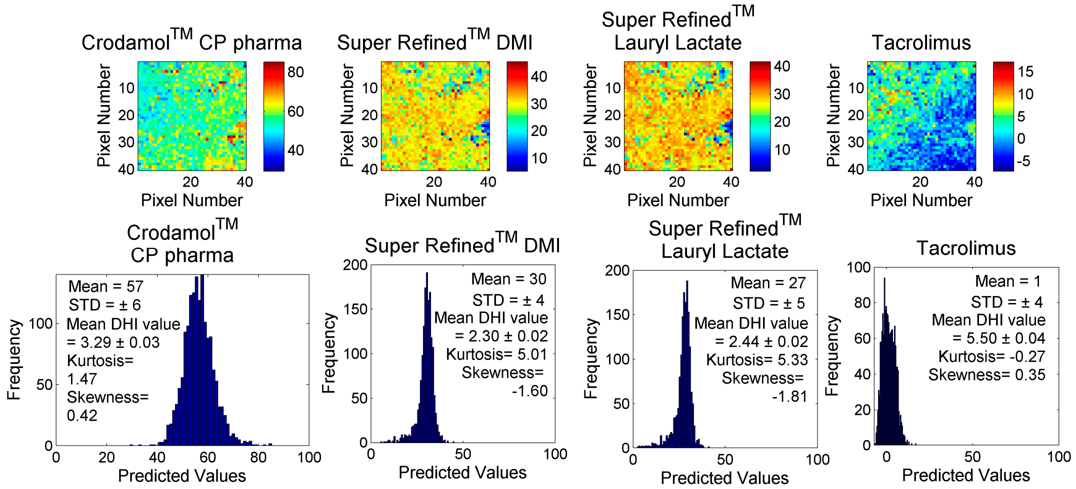

Figure S26. Chemical maps of the mixtures with the excipients Crodamol™ CP pharma (40 (% w/w)), SR™ DMI (25 (% w/w)) and SR™ Lauryl Lactate (25 (% w/w)) with the drug Tacrolimus, replicate 1

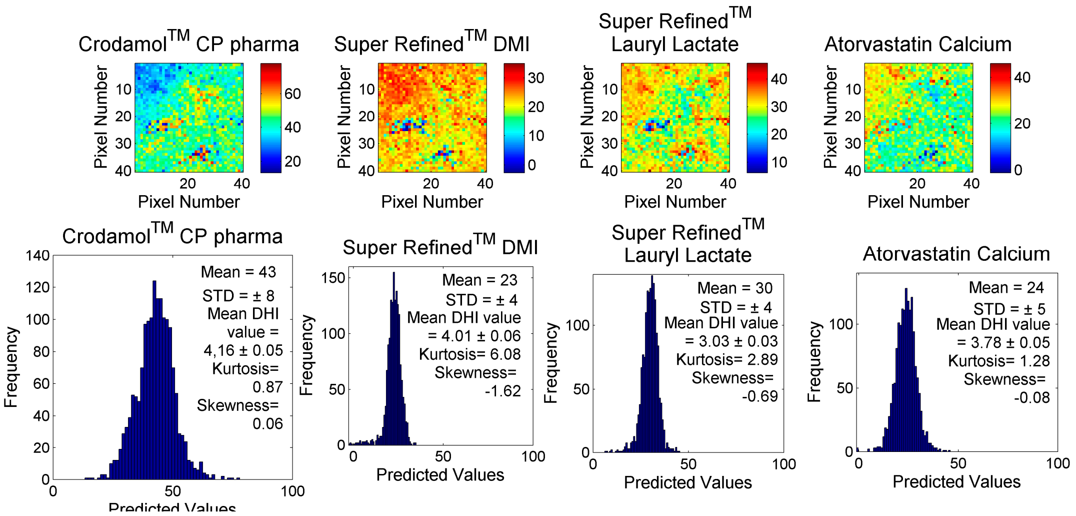

Figure S27. Chemical maps of the mixtures with the excipients Crodamol™ CP pharma (40 (% w/w)), SR™ DMI (25 (% w/w)) and SR™ Lauryl Lactate (25 (% w/w)) with the drug Atorvastatin Calcium, replicate 1

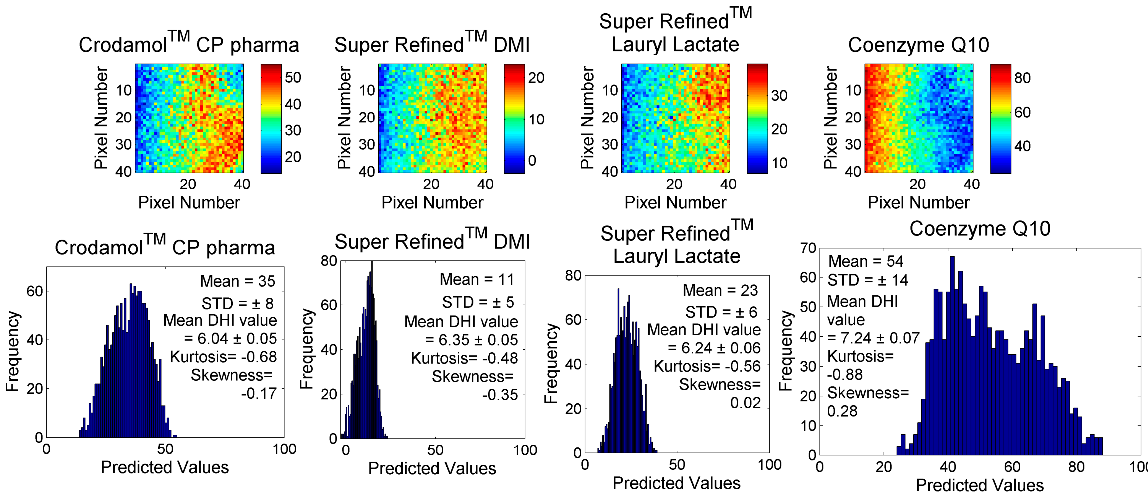

Figure S28. Chemical maps of the mixtures with the excipients Crodamol™ CP pharma (40 (% w/w)), SR™ DMI (25 (% w/w)) and SR™ Lauryl Lactate (25 (% w/w)) with the drug Coenzyme Q10, replicate 1

Table S3. Standard deviation, logP, and mean values of DHI and kurtosis for the evaluated drugs

Drug	logP	STD	DHI mean	Kurtosis mean
Caffeine	-0.070	16.83	2.81	-0.20
Acetaminophen	0.910	18.66	2.85	-0.62
Butamben	2.870	5.43	1.69	1.22
Resveratrol	3.100	5.91	4.19	0.36
Tacrolimus	3.300	4.06	4.89	0.73
Atorvastatin calcium	6.360	4.92	4.00	1.35
Coenzyme Q10	10.000	12.82	6.57	-0.55

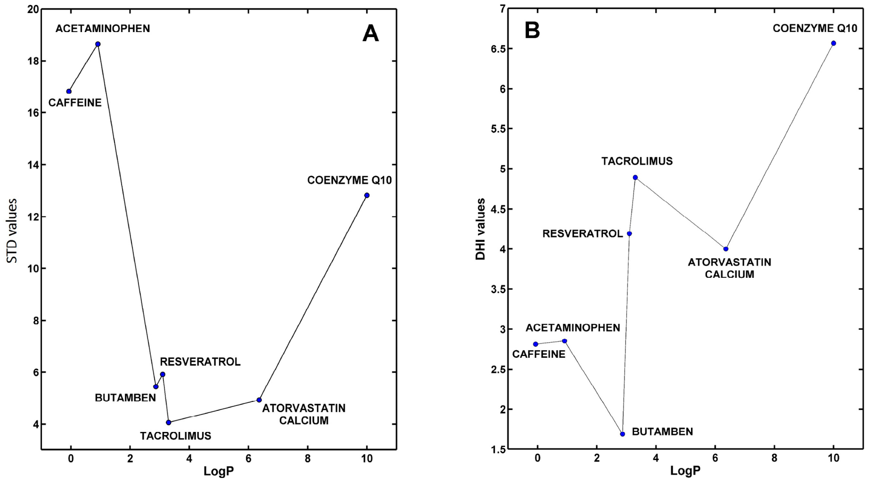

(**a**) (**b**)

Figure S29. Graph of LogP against (A) STD values and (B) DHI values for the evaluated drugs
